# Routine esophagram to detect early esophageal leakage after peroral endoscopic myotomy

**DOI:** 10.1055/a-2294-8607

**Published:** 2024-04-26

**Authors:** Elise M. Wessels, Sara Nullens, Barbara A.J. Bastiaansen, Paul Fockens, Gwen M.C. Masclee, Albert J. Bredenoord

**Affiliations:** 1 522567 Gastroenterology & Hepatology, Amsterdam UMC, Amsterdam, Netherlands; 2 60202 Department of Gastroenterology and Hepatology, University Hospital Antwerp, Edegem, Belgium; 3 60194 Department of Gastroenterology and Hepatology, GZA Hospitals, Wilrijk, Belgium; 4 Department of Gastroenterology, Amsterdam UMC, Duivendrecht, Netherlands

**Keywords:** Endoscopy Upper GI Tract, Motility / achalasia, POEM, Quality and logistical aspects, Performance and complications

## Introduction

Achalasia is a rare esophageal motility disorder that is characterized by absent or uncoordinated esophageal peristalsis and insufficient relaxation of the lower esophageal sphincter (LES), resulting in impaired propulsion of food through the esophagus. The most common symptoms include dysphagia, regurgitation of undigested food, chest pain, and weight loss 1 . Current treatment aims at reducing symptoms by lowering the pressure of the LES, therefore improving passage of food through the esophagus. Treatment options are pneumatic dilatation (PD), laparoscopic Heller myotomy (LHM) which is often combined with fundoplication, botulinum toxin injection, and peroral endoscopic myotomy (POEM) 2 .

Since the first successful POEM was reported in 2010, this procedure is increasingly being performed worldwide and it is now a key element in achalasia treatment 3 . POEM is a minimally invasive procedure in which muscle fibers of the distal esophagus and LES are cut endoscopically by making a submucosal tunnel toward the stomach.

Previous studies have shown that POEM is non-inferior to LHM in controlling symptoms of achalasia 4 5 . The long-term efficacy of POEM is higher than for PD with treatment success rates of 81% for POEM and 40% for PD after 5 years 6 .

POEM appeared to be safe when performed by experienced endoscopists 4 7 8 9 10 11 12 . However, the definition and classification of adverse events (AEs) varies between studies, and therefore, the number of AEs are wide-ranging from 0% to 35.8% 9 10 12 13 14 15 16 17 18 . Initially, all types of mucosal injury and gas-related events such as pneumoperitoneum and pneumomediastinum were reported as AEs. These events are commonly encountered on routinely performed post-procedure imaging after POEM, are often asymptomatic and usually do not affect patient outcome, hospital stay, or clinical management 19 . Therefore, routine imaging after POEM is not always recommended 20 21 22 23 24 . However, a clinical practice guideline for POEM states that upper gastrointestinal endoscopy has to be carried out after POEM to check for mucosal damage or hemorrhage and that a routine esophagram has to be performed to exclude esophageal leakage 25 . Another study concluded that routine postoperative computed tomography (CT) might be helpful in early detection of potential significant AEs, although most CT findings did not result in a need for additional treatment 26 .

Currently, there is no consensus about postoperative care after POEM and it is unknown to what extent performing routine postoperative imaging will result in early detection and better treatment of AEs 23 25 26 27 . The aim of this study was to evaluate incidence of early AEs following POEM and to assess whether post-procedure imaging by routine esophagram prevents serious AEs due to early detection of esophageal leakage after POEM.

## Patients and methods

### Study design

This retrospective cohort study was performed at the Amsterdam University Medical Center. Data were extracted from medical records of all consecutive patients who underwent POEM between August 2011 and December 2022. Diagnosis of achalasia, hypercontractile esophagus, or diffuse esophageal spasm had to be confirmed by manometry in order to be included in this study. Other inclusion criteria were a technical successful POEM procedure, at least 1 month of follow-up and aged ≥18 years. The first 25 procedures after introduction of POEM in our center were excluded, taking into account the learning curve for the procedure 28 . Patients undergoing gastric-POEM were also excluded from this study.

### POEM procedure

All POEM procedures were performed by two experienced interventional endoscopists (BB, PF) according to our protocol under general anesthesia and all patients received perioperative intravenous antibiotics. Carbon dioxide insufflation was routinely used in all patients. POEM started with submucosal injection of saline and indigo carmine halfway into the esophageal body followed by a 2-cm mucosal incision to enter the submucosal space. A submucosal tunnel toward the LES was created and colored saline was used to increase demarcation and to enlarge the submucosal working space. The submucosal tunnel was continued to approximately 3 cm beyond the LES. Afterward, myotomy of the circular muscle layer and part of the longitudinal muscle layer was performed. Once the myotomy was completed, the mucosal incision was closed with multiple endoclips.

### Post-procedure care

According to the protocol, standard observation after POEM was 1-night admission. Until July 2016, routine esophagram was performed 1 day after POEM in all patients to assess for signs of esophageal leakage. Patients had to drink ≥100 mL of liquid iodized contrast (Ultravist 300) in upright position. Radiographs of the esophagus were made to rule out leakage of contrast in the submucosal tunnel and/or in the mediastinum. Patients were discharged when no significant esophageal leakage was identified on routine esophagram and liquids were well tolerated. After July 2016, routine esophagram or chest CT in the first days post-POEM was only performed in case of symptoms suggestive for post-procedure AEs (e.g. uncontrolled retrosternal pain or fever) and patients were discharged when they were able to tolerate liquids 1 day post-POEM. After discharge, all patients followed a liquid diet for 1 week and a ground diet for another week. Standard endoscopy in the first postoperative days after POEM to assess mucosal damage, the location of the clips, and hemorrhage was not performed routinely. Repeat upper gastrointestinal endoscopy was only performed on indication.

### Outcome measures

The primary outcome was the number of early post-procedure AEs after POEM. Early post-procedure AEs were defined as any unfavorable event within 30 days after POEM and were graded according to the Adverse events Gastrointestinal Endoscopy (AGREE) classification 29 . The occurrence of early post-procedure AEs was routinely assessed shortly after the procedure, before discharge, after 2 weeks, and 1 to 3 months after POEM. This was documented in the medical record during follow-up. The number and grade of post-procedure AEs was compared between two cohorts of patients, with a focus on the AEs resulting from esophageal leakage. The first group included patients undergoing POEM before July 2016 with routine esophagram 1 day post-POEM. In the other group of patients, POEM was carried out after July 2016 and routine esophagram was not routinely performed in any of these patients. Secondary outcomes included hospital stay, signs of esophageal leakage on routine esophagram, and repeat endoscopy after POEM. Intraprocedure AEs were documented in the report of the POEM procedure. Pneumoperitoneum was reported as an intraprocedure AE when abdominal needle drainage was necessary or when the procedure was temporarily stopped because of a change in ventilation pressure. Bleeding was indicated as major in case of hemodynamic instability, blood transfusion, or prolonged hospitalization. Mucosal injury that occurred during POEM for which extra clips were needed was also considered to be an intraprocedure AE. All AEs were discussed by the adjudication committee, which consisted of the two experienced interventional endoscopists who performed the POEM procedures (BB, PF), to determine if the AEs could have been prevented or could have been less severe with early detection of esophageal leakage on routine esophagram.

### Statistical analysis

SPSS statistics version 28.0 was used for statistical analysis. Comparisons were made using Chi-square test, Fisher’s exact test or non-parametric testing (Mann-Whitney U test) where appropriate. Two-sided *P* <0.05 was considered to be statistically significant.

## Results

### Patient characteristics

**Fig. 1:**
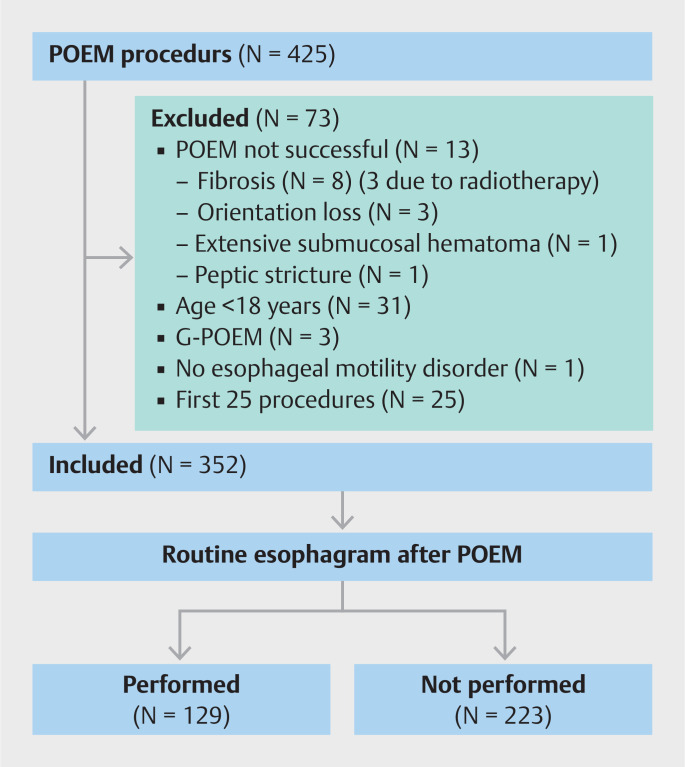
Flow chart. POEM, peroral endoscopic myotomy.

In total 425 patients underwent POEM between August 2011 and December 2022. Thirteen procedures were not successful due to submucosal fibrosis in the distal esophagus or around the LES, orientation loss, extensive submucosal hematoma, or peptic stricture. Of the remaining 412 patients, 31 patients were aged <18 years, three patients underwent gastric-POEM, and one patient had no esophageal motility disorder. The first 25 POEM procedures were excluded taking into account the POEM learning curve of the endoscopists. Hence, 352 patients were included in this study of whom 129 underwent POEM before July 2016 and 223 after July 2016 ( Fig. 1 ). All patients were routinely followed for at least 1 month and no patients were lost to follow-up. Patient characteristics are specified in Table 1 and procedure-related outcomes in Table 2 .

**Table:** **Table 1** Patient characteristics.

	**Total (N=352)**	**Routine esophagram (N=129)**	**No routine esophagram (N=223)**
Age, years, mean (SD)	47 (17)	47 (16)	47 (17)
Sex			
Female	170 (48.3)	61 (47.3)	109 (48.9)
Male	182 (51.7)	68 (52.7)	114 (51.1)
BMI, kg/m ^2^ , median (IQR)	24.0 (5.3)	23.6 (5.3)	24.2 (5.3)
ASA score			
I	126 (35.8)	65 (50.4)	61 (27.4)
II	193 (54.8)	56 (43.4)	137 (61.4)
III	33 (9.4)	8 (6.2)	25 (11.2)
Comorbidities			
Diabetes mellitus type II	23 (6.5)	6 (4.7)	17 (7.6)
Hypertension	54 (15.3)	20 (15.5)	34 (15.2)
OSAS	10 (2.8)	1 (0.8)	9 (4.0)
COPD or asthma	28 (8.0)	9 (7.0)	19 (8.5)
Thyroid disease	22 (6.3)	9 (7.0)	13 (5.8)
Malignancy (past or current)	14 (4.0)	3 (2.3)	11 (4.9)
Chronic inflammatory disease	16 (4.5)	5 (4.9)	11 (4.9)
Chronic renal failure	3 (0.9)	0 (0.0)	3 (1.3)
Barrett esophagus	1 (0.3)	1 (0.8)	0 (0.0)
Other cardiac or vascular disease	33 (9.4)	23 (10.3)	10 (7.8)
Other hematological disease	5 (1.4)	1 (0.8)	4 (1.8)
Other neurological disease	25 (7.1)	6 (4.7)	19 (8.5)
Previous surgery			
Abdominal surgery ^*^	67 (19.0)	22 (17.1)	45 (20.2)
Thoracic surgery	4 (1.1)	1 (0.8)	3 (1.3)
Esophageal motility disorder ^†^			
Achalasia	344 (97.7)	125 (96.9)	219 (98.2)
Type I	69 (19.6)	34 (26.4)	35 (15.7)
Type II	190 (54.0)	56 (43.4)	134 (60.1)
Type III	34 (9.7)	19 (14.7)	15 (6.7)
Non-specified	51 (14.5)	16 (12.4)	35 (15.7)
Jackhammer esophagus	3 (0.9)	0 (0.0)	3 (1.3)
DES	5 (1.4)	4 (3.1)	1 (0.4)
Previous treatment	259 (73.6)	83 (64.3)	176 (78.9)
PD	228 (64.8)	70 (54.3)	158 (70.9)
BTI	41 (11.6)	17 (13.2)	24 (10.8)
LHM	85 (24.1)	28 (21.7)	57 (25.6)
POEM	9 (2.6)	0 (0.0)	9 (4.0)
TBE before POEM			
Column height, cm, median (IQR)			
0 min	8.0 (6.1)	9.0 (7.2)	7.7 (5.4)
1 min	6.4 (5.3)	7.4 (5.2)	6.0 (4.8)
2 min	5.8 (4.7)	6.0 (5.7)	5.7 (4.3)
5 min	4.9 (4.8)	5.0 (5.0)	4.9 (4.6)
Max diameter, cm, mean (SD)	3.2 (1.2)	3.2 (1.2)	3.2 (1.2)
Sigmoid esophagus	10 (2.9)	2 (1.6)	8 (3.7)
Time between diagnosis and POEM, months, median (IQR)	14.0 (45.0)	9.0 (43.5)	15.0 (45.0)
Results are presented as n (%) unless otherwise stated. *Laparoscopic Heller myotomy not included. †Based on Chicago classification version 3.0. ASA, American Society of Anesthesiologists; BMI, body mass index; BTI, botulinum toxin injection; COPD, chronic obstructive pulmonary disease; DES, diffuse esophageal spasm; HIV, human immunodeficiency virus; IQR, interquartile range; LHM, laparoscopic Heller myotomy; OSAS, obstructive sleep apnea syndrome; PD, pneumatic dilatation; POEM, peroral endoscopic myotomy; SD, standard deviation; TBE, timed barium esophagram.			

**Table:** **Table 2** Procedure-related outcomes.

	**Total (N=352)**	**Routine esophagram (N=129)**	**No routine esophagram (N=223)**
Procedure time, minutes, median (IQR)	73 (39)	90 (37)	60 (32)
Length of myotomy, cm, median (IQR)	11 (3)	13 (4)	11 (3)
Selective circular	3 (3)	4 (4)	3 (3)
Full-thickness	9 (4)	9 (4)	8 (4)
Intraprocedure adverse events, number	41 ^*^	23	18
Pneumoperitoneum requiring abdominal needle drainage	18	15	3
Mucosal injury closed with clips	13	6	7
Second submucosal tunnel	4	0	4
Bleeding	6	2	4
Minor	4	1	3
Major	2	1	1
Number of days in hospital, days, median (IQR)	2 (0)	2 (0)	2 (0)
Routine esophagram post-POEM			
No esophageal leakage		124 (96.1)	
Signs of esophageal leakage		5 (3.9)	
Post-procedure adverse events ^†^	19 (5.4)	5 (3.9)	14 (6.3)
Grade I	10 (2.8)	2 (1.6)	8 (3.6)
Grade II	3 (0.9)	1 (0.9)	2 (0.9)
Grade IIIa	5 (1.4)	2 (1.6)	3 (1.3)
Grade IVa	1 (0.3)	0 (0.0)	1 (0.4)
Results are presented as n (%) unless otherwise stated.			

### Post-procedure adverse events

Post-procedure AEs within 30 days after POEM occurred in 19 patients (5.4%) of which 10 AEs were grade I (2.8%), three were grade II (0.9%), five were grade IIIa (1.4%) and one was grade IVa (0.3%) according to the AGREE classification 29 .

**Fig. 2:**
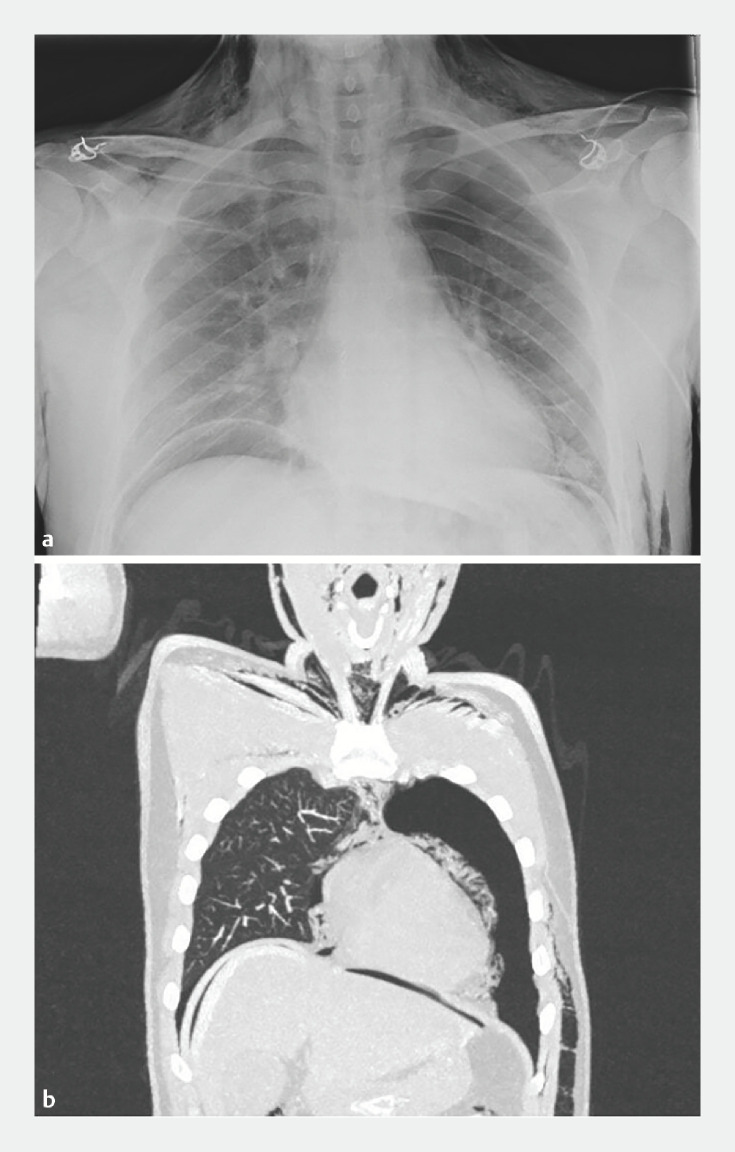
Pneumothorax, pneumoperitoneum, pneumomediastinum and subcutaneous emphysema after POEM. **a** Chest X-ray. **b** Chest CT. POEM, peroral endoscopic myotomy; CT, computed tomography.

**Fig. 3:**
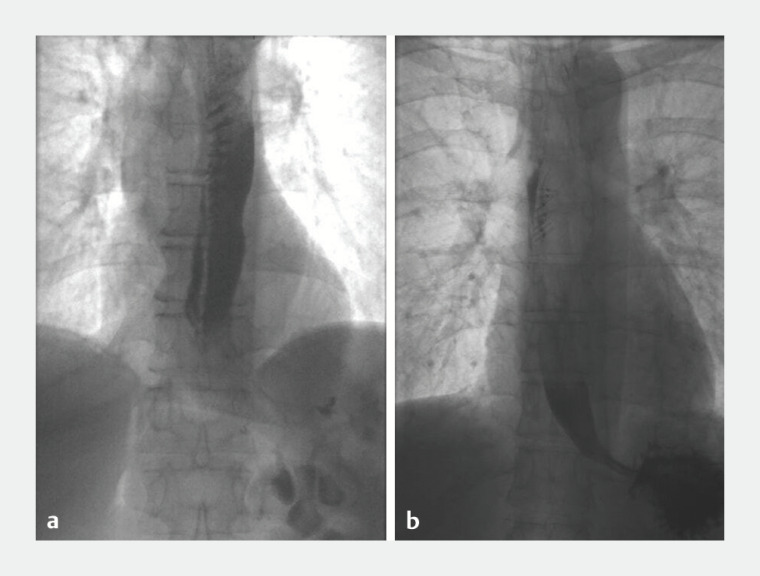
Submucosal esophageal leakage on routine esophagram. **a** Deep leakage into the submucosal tunnel toward the stomach. **b** Superficial leakage limited to the mucosal incision.

Supplementary Table 1 provides an overview of all post-procedure AEs. One patient had symptomatic pneumothorax, pneumoperitoneum, pneumomediastinum, and subcutaneous emphysema after POEM ( Fig. 2 ). In this patient, the procedure was inadvertently started with room air insufflation instead of carbon dioxide. This was noticed 30 minutes after introduction of the endoscope and insufflation was at that point switched to carbon dioxide. After the procedure, no chest or abdominal drainage or other intervention was necessary and the patient was hemodynamically stable and received extra oxygen for 3 days. Pain was controlled with opioids for 4 days and the patient was discharged after 7 days. Five patients underwent repeat endoscopy after POEM (grade III). In two of these patients, POEM was performed before July 2016 and those patients underwent repeat endoscopy because submucosal esophageal leakage was seen on routine esophagram and was closed with extra clips ( Fig. 3 ). One of these patients had deep submucosal leakage that extended through the submucosal tunnel up to the stomach but did not enter the mediastinum. The other patient had superficial esophageal leakage that was limited to the level of the mucosal incision ( Fig. 3 ). Because of persistent esophageal leakage on the esophagram the day after repeat endoscopy in both patients, another repeat endoscopy was carried out in which two extra clips were used to close the incision and a duodenal feeding tube was placed. Both patients were asymptomatic before repeat endoscopy and no opioids were necessary for retrosternal pain. One of these patients received antibiotics for 2 weeks because of fever measured once after repeat endoscopy. The patients were discharged after 5 and 7 days.

One patient was readmitted to the intensive care unit (ICU) because of respiratory insufficiency 3 days after POEM and required ICU observation with oxygen support (grade IVa). CT showed significant pleural effusion and debris in the right main bronchus without signs of esophageal leakage. Upper endoscopy was performed and did not show leakage or perforation and all clips were in place. The pleural effusion was punctured, but no bacteria were identified on culture. The patient was stable with high flow oxygen and antibiotics and able to be discharged from the hospital after 9 days, 6 of which were in the ICU. Further recovery was unremarkable. No AEs resulted in death.

### Routine esophagram

**Fig. 4:**
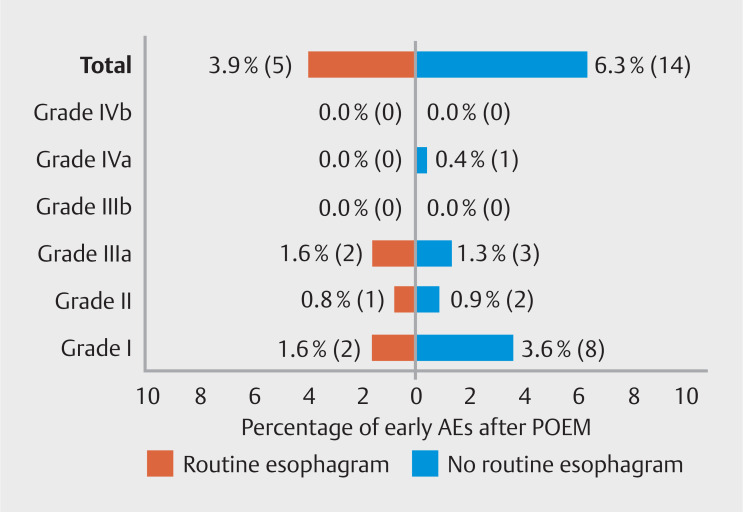
Percentage (number) of patients with an early AE after POEM. There was no difference in the number and severity of AEs among patients with and without routine esophagram. AE, adverse event. POEM, peroral endoscopic myotomy.

Routine esophagram was standard performed 1 day after POEM in 129 patients of whom five had post-procedure AEs (n=5/129, 3.9%). Two were classified as grade I (n=2/129, 1.6%), one as grade II (n=1/129, 0.8%), and two as grade IIIa (n=2/129, 1.6%). Fourteen post-procedure AEs (n=14/223, 6.3%) occurred in the other group in which routine esophagram was not standard performed ( Fig. 4 ). Of these AEs, eight were grade I (n=8/223, 3.6%), two were grade II (n=2/223, 0.9%), three were grade IIIa (n=3/223, 1.3%), and one was grade IVa (n = 1/223, 0.4%). Overall, the number and severity of the AEs was equal between patients with and without routine esophagram. Most importantly, no severe complications due to esophageal leakage (e.g. sepsis or mediastinitis) were observed.

In five patients, possible signs of esophageal leakage were reported (n=5/129, 3.9%) of whom two patients underwent a subsequent endoscopy to close the potential leakage with additional clips. The esophageal leakage on routine esophagram of the other three asymptomatic patients was questionable because it was very minimal and limited to the level of the mucosal incision. These patients were treated conservatively without repeat endoscopy; therefore, this was not classified as an AE.

If routine esophagram had been performed after July 2016, esophageal leakage may have been seen in one patient with retrosternal pain in whom partial dehiscence of the mucosal incision was observed during repeat endoscopy 5 days after POEM. However, no endoscopic intervention was needed and the patient recovered with conservative treatment. The adjudication committee decided that a routine esophagram would only have detected the esophageal leakage earlier, but management would not have changed because the esophageal leakage was limited to the mucosal incision and no endoscopic intervention was needed. Adjudication concluded that none of the other AEs could have been detected earlier by routine esophagram because no other AE was associated with esophageal leakage.

## Discussion

This study demonstrated that POEM is safe and that the number and severity of AEs is not different whether or not an esophagram is performed routinely 1 day after POEM. Even more importantly, after abolishing routine esophagram from the protocol ,no AEs associated with esophageal leakage led to severe complications that necessitated additional or more comprehensive interventions. Therefore, routine esophagram after POEM could not have prevented the occurrence of any AEs and would not have impacted management to a significant extent.

Post-procedure AEs within 30 days after POEM occurred in 19 of 352 patients (5.4%). Intraprocedure AEs occurred more frequently than post-procedure AEs. In total 41 intraprocedure AEs occurred in 38 patients (11.6%). These procedure-related events were already managed during POEM and did not influence patient outcome or hospital stay. Pneumoperitoneum for which abdominal needle drainage was necessary occurred primarily before July 2016. A possible explanation for this might be that it became common practice to increasingly widen the submucosal tunnel, allowing carbon dioxide to escape more easily.

The number of AEs in this study was slightly lower compared with the study of Haito-Chavez (2017), who reported that AEs after POEM occurred in 7.5% of the 1826 included patients. However, that percentage comprised intraprocedure AEs as well as post-procedure AEs. In total, 156 AEs occurred, of which 89 were during the procedure and 67 post-POEM 7 . The difference in AE rates might also be explained by the different classification that was used. For example, post-procedure medical consultation without presentation in the hospital and without intervention is seen as mild AE according to the ASGE lexicon’s severity grading system and as no AE when using the AGREE classification 29 30 . In a study comparing POEM and LHM in 221 patients, serious AEs occurred in 2.7% and 7.3% of patients undergoing POEM and LHM, respectively. The number of non-serious AEs was 12 (11%) after both procedures. Intraprocedure AEs were also included in this number 4 . Another randomized controlled trial by Ponds et al. (2019) reported two serious AEs after PD and absence of AEs after POEM. Non-serious AEs occurred in 67% and 22% of the patients after POEM and PD, respectively. However, 37 of 42 non-serious AEs after POEM were ascribed to the presence of reflux esophagitis and reflux symptoms after follow-up of more than 30 days 8 .

Currently, post-procedure care varies per hospital and routine esophagram 1 day post-POEM still is often performed 27 31 . Five of 154 patients had signs of esophageal leakage on routine esophagram, of which two were indicated as clinically relevant and repeat endoscopy was performed to close the leakage with additional clips. After July 2016, routine esophagram was no longer performed and no serious complication occurred due to esophageal leakage, such as mediastinitis or an abscess, which could have been prevented by performing routine esophagram 1 day after POEM. After July 2016, more patients were observed 1 day longer because of symptoms suggestive for esophageal leakage, but this did not influence hospital stay. In these patients, CT was performed, which did not show esophageal leakage, and symptoms improved the next day in all patients. Repeat endoscopy was carried out three times after July 2016 because of retrosternal pain 4 and 5 days after POEM in two patients and melena 3 weeks after POEM in one patient. For these patients, extra clips or other additional endoscopic intervention were not needed during repeat endoscopy. These patients fully recovered with conservative management. Although severe complications resulting from esophageal leakage did not occur in any of the patients in our study, these complications can be life-threatening. Therefore, we recommend performing CT or upper endoscopy as a valid surrogate for routine esophagram after POEM when symptoms suggest that esophageal leakage is present.

A previous study with 78 patients evaluating the need for routine esophagram 1 day after POEM reported a high sensitivity of 100% and a low specificity of 45%. Abnormal findings on routine esophagram were present in 72% of the patients and usually had no clinical significance 21 . Another study in which routine esophagram after POEM was performed in 170 patients found abnormalities with limited clinical significance in most patients. Routine esophagram correctly identified esophageal leakage in two patients, but the findings were false-negative in two other patients and false-positive in one patient. The authors concluded that routine esophagram alone was not reliable enough to identify AEs 23 . Some studies are in favor of performing CT to detect AEs and to initiate prompt intervention 26 32 , but others do not recommend routine postoperative CT because of limited clinical significance 20 22 . Abnormal findings, such as pneumoperitoneum and subcutaneous emphysema, are often seen on radiographic imaging after POEM and do not influence clinical management or patient outcome 19 20 21 23 26 32 33 . Therefore, findings for which intervention is not necessary should not be regarded as AEs 19 33 . Performing routine esophagram 1 day post-POEM to assess delay in passage of contrast does not predict long-term efficacy of POEM, and thus, is not useful for that purpose either 34 35 .

This is the first study comparing AEs after POEM in patients with and without routine esophagram 1 day post-POEM. The year in which POEM was carried out differed between the two groups in our study, but other factors remained the same and a difference in post-procedure care would probably not have influenced the number or severity of post-procedure AEs. No changes have been made to the antibiotics prophylaxis and the post-procedure fasting protocol. It is plausible that the level of experience of the endoscopist was higher after July 2016, which might also explain the shorter procedure time after July 2016. A systematic review concluded that proficiency in performing POEM is obtained after 25 procedures. Although no post-procedure AEs occurred in the first 25 procedures, they were excluded for the above-mentioned reason from further analysis. A limitation of the study is that we do not know whether the two patients in which esophageal leakage was seen on routine esophagram and subsequent repeat endoscopy was carried out would have become symptomatic when repeat endoscopy with additional clip placement was not performed, and thus, whether potentially more serious AEs may have been prevented. This study is also limited by the absence of esophageal perforations in patients undergoing POEM after July 2016. However, this illustrates that esophageal perforation after POEM is uncommon and that POEM is safe. Finally, this was a retrospective cohort study performed in one center and the best study design to assess the need for routine esophagram after POEM would be a randomized controlled trial, but that would require very large numbers of patients, which does not seem feasible for this rare disease. The prospective collected data in this study and the accurate registration of AEs resulted in a high-quality database with limited missing data and would seem a good alternative to the above. A relatively large number of patients were included in this study and no patients were lost to follow-up. Nevertheless, due to the small number of AEs, we could not perform multivariate logistic regression analysis to assess possible predictors of AEs occurrence.

## Conclusions

In conclusion, the results of this study show that POEM is safe and routine esophagram 1 day after POEM is unlikely to be of additional value in preventing serious AEs resulting from esophageal leakage. AEs occurring after July 2016 could not have been prevented by performing routine esophagram 1 day after POEM, therefore, we recommend performing postoperative imaging only in patients who have symptoms suggestive of post-procedure AEs. This approach will reduce costs and radiation exposure and allow for more rapid discharge of patients after POEM.

## Supplementary Material

Supplementary Material Zusatzmaterial
