# Supplementary material for: Routine esophagram to detect early esophageal leakage after peroral endoscopic myotomy
Source: Endosc Int Open. 2024 Apr 26;12(4):E604–12. doi: 10.1055/a-2294-8607 (PMC11052647; doi:10.1055/a-2294-8607)
Supplement: Supplementary file 1 — Supplementary Material [file 10-1055-a-2294-8607_22953812.pdf]

Thieme

Supplementary material

Supplementary Table 1 Overview of post-procedure adverse events.

Patient	Post-procedure AE	Diagnostics	Hospital stay	AGREE-classification	Year of POEM	Days between POEM and AE
1	Retrosternal pain with opioids for pain control	Routine barium esophagram: no esophageal leakage	Prolonged > 24 hours<br>Total 4 days	Grade II	2013	1
2	No symptoms. Repeat endoscopy because of signs of esophageal leakage, three extra clips were placed at the incision. The next day persistent leakage for which a duodenal feeding tube was placed. Antibiotics were given because of fever measured once after repeat endoscopy	Routine barium esophagram: submucosal leakage<br>Second barium esophagram: persistent leakage	Prolonged > 24 hours<br>Total 7 days	Grade IIIa	2015	1
3	No symptoms. Twice repeat endoscopy because of signs of esophageal leakage. A total of five extra clips were placed at the incision. A duodenal feeding tube was placed for 3 days because of persistent leakage	Routine barium esophagram: submucosal leakage<br>Second barium esophagram: persistent leakage	Prolonged > 24 hours<br>Total 5 days	Grade IIIa	2016	1
4	Retrosternal pain with opioids for pain control	Routine barium esophagram: no esophageal leakage	Prolonged < 24 hours<br>Total 3 days	Grade I	2016	1
5	Longer observation because of intraprocedure bleeding. Hemodynamically stable. No blood transfusion was needed	Routine barium esophagram: no esophageal leakage	Prolonged < 24 hours<br>Total 3 days	Grade I	2016	1
6	Retrosternal pain with opioids for pain control. Antibiotics were given for 2 days until perforation was ruled out by upper endoscopy	CT: pneumoperitoneum, no signs of esophageal leakage, possible microperforation<br>Upper endoscopy: no abnormalities	Readmission 48 hours	Grade IIIa	2017	4

Supplementary material

7	Melena 3 weeks after POEM due to ulcer in the cardia where previously mucosal injury was clipped during POEM. Hemodynamically stable. Recovered with conservative treatment	Upper endoscopy: ulcer at the place of previous mucosal injury which was clipped during POEM	Not prolonged<br>Upper endoscopy at outpatient clinic	Grade IIIa	2017	21
8	Longer observation because of low blood pressure	None	Prolonged < 24 hours<br>Total 3 days	Grade I	2017	0
9	Retrosternal pain with non-opioids for pain control	Chest X-ray: no abnormalities	Prolonged < 24 hours<br>Total 3 days	Grade I	2017	0
10	Pneumoperitoneum, pneumomediastinum, subcutaneous emphysema and pneumothorax after inadvertently using room air instead of carbon dioxide during POEM. Drainage was not needed and opioids were given for pain control. Hemodynamically stable	Chest X-ray and CT: pneumoperitoneum, pneumomediastinum, subcutaneous emphysema and pneumothorax	Prolonged > 24 hours<br>Total 7 days	Grade II	2017	0
11	Retrosternal pain with opioids for pain control	CT: no abnormalities	Visit emergency department without re-admission	Grade I	2018	1
12	Oral antibiotics for 5 days because of pneumonia	Chest X-ray: pneumomediastinum, consolidation	Not prolonged<br>Total 2 days	Grade II	2019	1
13	Longer observation because of intraprocedure bleeding and difficult closure of mucosal incision. Nasogastric tube was placed until esophageal leakage was ruled out by CT. No symptoms	CT: pneumomediastinum, pneumoperitoneum, no esophageal leakage	Prolonged < 24 hours<br>Total 3 days	Grade I	2019	0
14	Retrosternal pain with non-opioids for pain control	CT: pneumoperitoneum, no esophageal leakage	Prolonged < 24 hours<br>Total 3 days	Grade I	2020	1
15	Low-grade fever and retrosternal pain with non-opioids for pain control. Readmission for observation and the patient recovered with conservative treatment	CT: pneumoperitoneum, no esophageal leakage	Visit emergency department with re-admission for 1 night/day	Grade I	2021	2

Supplementary material

16	Abdominal pain and respiratory insufficiency three days after POEM. Pneumonia with pleural effusion. ICU admission with high flow oxygen, ceftriaxone, metronidazole and pleural drainage (exudate, no bacteria)	CT: significant pleural effusion, no esophageal leakage, minimal pneumomediastinum, pneumoperitoneum, atelectasis, debris in right main bronchus<br>Upper endoscopy: no perforation	Re-admission 9 days (6 days ICU)	Grade IVa	2021	3
17	Five days after POEM retrosternal pain with non-opioids for pain control. Duodenum feeding tube was placed for 25 days because of dehiscence of the mucosal incision	Upper endoscopy: dehiscence of mucosal incision with closed tunnel after removing clip, no extra clips placed, duodenum feeding tube placed	Re-admission for 1 night	Grade IIIa	2021	5
18	Retrosternal pain after 3 days with non-opioids for pain control	CT: pneumoperitoneum, no esophageal leakage	Visit emergency department without re-admission	Grade I	2022	3
19	Longer observation because of retrosternal pain 1 day post-POEM with non-opioids for pain control. After 1 week fever measured once at home and no change in retrosternal pain. Pain well controlled with non-opioids	CT after 1 day: no esophageal leakage, little intramural contrast<br>CT after 1 week: no esophageal leakage, lung nodule 5 mm (follow up after 6 months)	Prolonged < 24 hour<br>Total 3 days<br>Visit emergency department without re-admission	Grade I	2022	1 and 7

AE, adverse event; AGREE, Classification for Adverse events Gastrointestinal Endoscopy; ICU, intensive care unit; POEM, peroral endoscopic myotomy.
